# Time-of-Day Effects on Anaerobic Power and Concentration of Selected Hormones in Blind Men

**DOI:** 10.3390/ijerph18179353

**Published:** 2021-09-04

**Authors:** Tomasz Pałka, Przemysław Pajor, Anna Katarzyna Tyka, Wanda Pilch, Agata Cebula, Aneta Teległów, Marek Strzała, Marcin Maciejczyk

**Affiliations:** 1 Department of Physiology and Biochemistry, Faculty of Physical Education and Sport, University of Physical Education, 31-571 Kraków, Poland; tomasz.palka@awf.krakow.pl; 2 Doctoral Studies, University of Physical Education, 31-571 Kraków, Poland; fizjoterapiapajor@gmail.com; 3 Department of Recreation and Biological Regeneration, Faculty of Tourism and Leisure, University of Physical Education, 31-571 Kraków, Poland; anna.tyka@awf.krakow.pl; 4 Department of Cosmetology, Faculty of Rehabilitation, University of Physical Education, 31-571 Kraków, Poland; wanda.pilch@awf.krakow.pl; 5 Department of Biological Regeneration and Posture Correction, Faculty of Physical Education and Sport, University of Physical Education, 31-571 Kraków, Poland; agata.cebula@awf.krakow.pl; 6 Department of Clinical Rehabilitation, University of Physical Education, 31-571 Kraków, Poland; aneta.teleglow@awf.krakow.pl; 7 Department of Water Sports, Faculty of Physical Education and Sport, University of Physical Education, 31-571 Kraków, Poland; marek.strzala@awf.krakow.pl

**Keywords:** hormone, anaerobic power, physical fitness, blind men, melatonin, Wingate test

## Abstract

Knowledge of the circadian rhythm of the blind person and diurnal changes in anaerobic power and hormones concentration can create the possibility of individualising physical training. The aim of the study was to examine the time-of-day effects on anaerobic performance and the concentration of selected hormones. The measurements were performed at two different times of the day (10:00 a.m., 10:00 p.m.) in blind men at the age of 20–25 years old. The experiment group was chosen by using repeated hormonal tests four times a day so that each selected patient had a sleep/wake cycle even of 24 h. Anaerobic peak power and total work were tested in an anaerobic sprint test, and the concentration of growth hormone, testosterone, cortisol, and melatonin was determined. In blind men, the hormonal response was not driven by the photoperiod as in the control group. In the blind group, at 10:00 p.m., anaerobic peak power and total work results were significantly higher than at 10:00 a.m. and negatively correlated with melatonin levels. No such correlation was found in the control group.

## 1. Introduction

Studies on human circadian rhythm have been of interest to many researchers [1,2,3,4,5,6]. Due to their nature, it is necessary to carry out research at practically all times of the day, taking seasonal rhythms into account. They are difficult to implement and burdensome. Therefore, such studies [2,3,4,5,6] are sporadically carried out and are most often limited to the study of circadian rhythms in people with sleeping disorders, less frequently in non-disabled people, including athletes. There is a small percentage of such research among blind people. In recent years, the percentage of blind individuals practicing sport has been steadily growing, which further justifies the need for conducting such tests.

When people are completely isolated from environmental signals, their circadian rhythms are activated with an almost 24 h cycle generated by the internal biological clock. It is emphasised that only an intact visual system may be necessary to synchronise the circadian system [7]. Most people who are blind have “free” circadian rhythms, i.e., they are not synchronised with environmental time signals and oscillate in a cycle that is slightly longer than 24 h. This condition causes recurrent insomnia and drowsiness during the day when rhythms fall out of the normal 24 h cycle phase, which may affect the physical performance of the body. Due to the low number of subjects, the prevalence and clinical significance of studies on circadian rhythm disturbances among blind people remains uncertain [7]. The high incidence of abnormal circadian rhythms in blind people underlines the importance of the light-dark cycle as a significant environmental synchroniser for the human circadian system. The activity of the human is largely regulated by circadian rhythm, with most physiological responses showing cyclic circadian variations. They are, in particular, subject to the processes associated with the endocrine system, most strongly modifying metabolism and activity of the autonomic nervous and digestive systems, thermoregulation, blood, circulatory and immune systems, as well as psychomotor performance modifying sleep and wakefulness processes [2,4,5,6,8]. The melatonin (M) is the main coordinator of harmonious work concerning the whole system. In non-disabled people, after 7:00 p.m., an increase in M concentration in the plasma can be observed, with peak values recorded from 10:00 p.m. to 2:00 a.m. In non-disabled persons, the biological clockwork has already been well-understood, affecting the circadian variability of physical performance indices in relation to the change in rectal temperature (Tre) and the secretion of selected hormones (growth hormone (GH), testosterone (T), cortisol (C) and M). The biological clock, influencing the change in hormone secretion, modifies the physiological responses determining the exercise capacity at different times of the day. The round-the-clock variability of some physiological markers, and in the case of particular hormones, is consistent with the body’s ability to vary in intensity and duration of physical work [1,4], which is important not only for athletes but also for people doing shift work or changing time zones. Cyclical sleeping disorders in the blind without light perception are quite common, and problems with sleep occur in up to 66% of people, with more frequent occurrences of circadian rhythm disorders in people who are completely blind than in those with slight vision [9].

On the basis of comparative analysis among blind and non-blind men, it will be possible to determine the correlation between hormones concentrations at different times of the day and anaerobic performance. This is particularly important for professional athletes with varying degrees of disability, including blind people, as it creates the possibility to individualise physical training loads and plan the start of sports competitions in different time zones.

In anaerobic tests conducted among sighted men, the results of peak power (PP) and total work (TW) reach the highest values in the afternoon (at 6:00 p.m.) and lower ones in the morning at 6:00 a.m. [10]. The highest testosterone concentration is recorded between 8:00 a.m. and 9:00 a.m., while the lowest can be noted in the evening, while for cortisol, it is at 9:00 a.m. and 2:00 a.m., respectively. Cortisol and testosterone modify the catabolic and anabolic processes [11].

The aim of the study was to examine the effect of time of day on anaerobic power (PP and TW) and the concentration of GH, T, C, and M hormones and to determine the relationship between them in blind and sighted men. We hypothesised that changes in hormone concentrations and anaerobic power in non-sighted men would be different from those in sighted men and unrelated to biological (chronological) rhythm.

## 2. Materials and Methods

### 2.1. Study Design

All the blind participants enrolled in the study were born with optic nerve damage and had no perception of light from birth (inclusion criteria). Healthy, not involved in competitive sports peers were recruited into the control group. Participants from the control group were physically active (light to moderate exercise ≤ three times per week), but their physical activity was not regular but only occasional.

A total of 16 blind men (BG) aged 20–25 years and the same number of sighted men (control group: CG) were selected for the study via deliberate selection. Each chronobiological experiment in completely blind patients required a thorough individual examination of circadian rhythms prior to the experiment. In this study, the BG group was chosen using repeated hormonal measurements, 4 times a day, to confirm that each participant had a sleep/wake cycle equal to 24 h. The criterion for choosing men for CG was the lark chronotype. The scope of our research has been limited to the study of only selected variables at two times of the day: 10:00 a.m. and 10:00 p.m.

Participants took part in two exercise tests. The first (graded test) assessed aerobic capacity (maximal oxygen uptake (VO_2_max)). On the basis of the results of this test, the intensity of the warm-up for the anaerobic test was determined. The anaerobic performance was assessed in a short (10 s) supramaximal sprint performed on a cycloergometer. The blood samples for hormones measurement were taken before the anaerobic test. The physician qualified the participants for the tests. Each group was randomly divided into 8, 2-person pairs so as to start basic research at different times of the day (cross-sectional study). The study was carried out two times of the day, at 10:00 a.m. and 10:00 p.m.

During the study period, the men did not participate in sports training and did not use any pharmacological agents that could affect the results of the study. The participants were asked to maintain their current diet and physical activity and not to use any wellness treatments (e.g., hydrotherapy, sauna, massage, cold-water baths, and local cryostimulation). For 2 days before, on the day of the test, the participants did not perform any intense physical efforts. They did not consume alcohol, caffeine-containing products, or other stimulants. The study protocol was approved by the Bioethical Commission of the Regional Medical Chamber in Krakow, Poland (No. 144/KBL/OIL/2011). All methods were performed in accordance with relevant guidelines/regulations. All participants were acquainted with the purpose and course of research. They also provided their written informed consent to participate in the project.

### 2.2. Somatic Measurements

The following anthropometric measurements were measured: body height (BH), body mass (BM), body fat percentage (PF), and lean body mass (LBM). Body mass and composition were established using the method of bioelectrical impedance, with a body composition analyser (IOI 353, Jawon Medical, Seoul, Korea), whereas body height was assessed using a stadiometer (Seca, Hamburg, Germany) with 1 mm accuracy.

### 2.3. Incremental Test

The graded test, in an air-conditioned laboratory at an ambient temperature of 21 ± 1.5 °C and 40%–60% relative humidity, included a graded effort until refusal, preceded by a 5-min warm-up at 60 W, followed by a 30 W increase in power every 3 min. The trial was performed until the participant reported volitional exhaustion and refused to continue the test. The pedalling cadence was 60 ± 3 rpm^−1^. At 30 s intervals, the oxygen intake (VO_2_) was recorded. The highest noted VO_2_ in the test was considered as VO_2_max. Parameters in the graded test were analysed in 30 s sequences using the Cortex MetaLyzer ergospirometer (Leipzig, Germany). The graded test was conducted on a Jaeger (Wuerzburg, Germany) cycloergometer.

### 2.4. Anaerobic Supramaximal Test

The men performed a 10 s supramaximal anaerobic exercise that was preceded by a 5 min warm-up at an intensity of 50% ± 5% VO_2_max, with a pedalling rate of 60 rpm, with three 5 s pedal accelerations at 2, 4 and 5 min. The load in the test amounted to 7.5% BM. The participant’s task was to reach the maximal pedalling rhythm in the shortest possible time and to maintain it for as long as possible. During the exercise, peak power and total work were recorded using software (MCE 5.2, JBA Staniak, Warsaw, Poland). The test was carried out on a cycloergometer (Monark 834E, Stockholm, Sweden).

### 2.5. Biochemical Procedures

In venous blood samples (Vacutainer system) taken before the anaerobic test, the concentration of hormones: cortisol, testosterone, growth hormone, and melatonin were determined. The blood intended for serum obtainment was centrifuged after 20 min of clotting at room temperature. Venous blood was centrifuged for 15 min at 4 °C, RCF 1000× *g* (MPW 351R, Warsaw, Poland). Serum samples were stored until analysis at −70° CT. The hormones were measured in blood serum by an enzyme-linked immunosorbent assay (ELISA) using high-sensitivity reagent kits (DRG Instruments Gmbh, Marburg, Germany): cortisol—EIA-1887 competitive binding, testosterone—EIA-1559 competitive binding, melatonin—EIA-1431 sandwich ELISA, growth hormone—EIA-1787 competitive binding. Concentrations of all hormones were measured once using a Chromate 4300 Microplate Reader (Awareness Technology, Palm City, FL, USA) at 450 nm. Standard curves for individual hormones were made twice. The sensitivities for the assays were as follows: cortisol = 1.3 ng/mL, growth hormone: 0.5 ng/mL, testosterone: 0.083 g/mL, melatonin: 1.6 ng/mL, respectively.

Percentage changes in plasma volume after exercise were calculated on the basis of haemoglobin concentration and haematocrit value according to the equation by Dill and Costill [12] modified by Harrison et al. [13]. Measured post-exercise melatonin, testosterone, cortisol, and growth hormone concentrations were adjusted according to the formula by Kraemer and Brown [14].

### 2.6. Statistical Analysis

Statistical analysis was carried out using the “windows R-3.5.1 environment” statistical package. In order to describe the collected quantitative data, the following descriptive statistics were used: arithmetic mean (M), standard deviation (SD). In the case of qualitative variables, grouping was carried out into separate classes, for which the number and percentages were calculated. The following tests were used in statistical analysis: The Shapiro–Wilk test. With this test, the assumption of normality of distribution of the quotient variables in each of the studied groups was examined. The results of this test indicated that the distribution of the majority of variables in the studied groups is not near normal distribution. General linear model (GLM-MANOVA) for repeated measurements in order to investigate the changes in variables studied over time (time (pretest vs. posttest) × group (blind vs. control group)). This model can be used for variables, the distribution of which is not close to normal. To determine the strength of the effect, partial eta squared (*η^2^_P_*) was calculated, the values of which >0.01, 0.06, and 0.14 correspond to the low, moderate, and high effect force [15]. The Mann–Whitney U Test, additionally, by means of this non-parametric test, the differences between groups (blind vs. control) were examined. The use of this test allowed to reduce the chance of making the first type of mistake (incorrect rejection of the null hypothesis). Spearman’s signed-rank correlation test was used to examine the relationship between two variables in the tested groups. In all analyses, significant effects were assumed to be those for which the probability value of *p* was lower than the assumed significance level of α = 0.05 (*p* < 0.05).

## 3. Results

### 3.1. Participants’ Characteristics

The average values of morphological indices in the BG and CG groups were in the following order: BH—173.8 ± 4.99 cm and 174.9 ± 3.87 cm, BM—74.23 ± 12.04 kg and 76.65 ± 4.26 kg, PF—21.92% ± 4.12% and 9.84% ± 2.67%, LBM 59.10 ± 5.80 kg and 70.30 ± 5.05 kg, respectively. The maximal oxygen uptake was 35.89 ± 5.95 mL kg^−1^ min^−1^ in BG and 46.45 ± 3.71 mL kg^−1^ min^−1^ in CG.

### 3.2. Peak Anaerobic Power and Total Work

In the CG at 10:00 a.m., peak power (12.58 ± 0.7 W kg^−1^) was, on average, 2.59 W kg^−1^ higher compared to the BG (9.99 ± 1.88 W kg^−1^). The observed differences are statistically significant (*Z* = 4.523, *p* < 0.001). The results of the total work in the CG for tests at 10:00 a.m. (111.38 ± 4.95 J kg^−1^) were, on average, 24.25 J kg^−1^ higher compared to the BG (87.13 ± 14.08 J kg^−1^). The TW results indicated that the observed differences are also statistically significant (*Z* = 4.609; *p* < 0.001) (Table 1).

In both groups, the results showed a statistically significant change in PP in the studied circadian cycle (*F* = 6.679; *η^2^_P_* = 0.182; *p* = 0.015). At 10:00 p.m., an increase in this parameter was observed in men compared to the measurement at 10:00 a.m. The observed differences were mainly influenced by the results of subjects from the BG for whom an increase in PP by 0.6 W kg^−1^ was noted, whereas in those for the CG, there were no significant changes in this parameter (interaction “time × group”: (*F* = 5.945; *η^2^_P_* = 0.165; *p* = 0.021)).

The results of statistical analysis performed for both groups indicated statistically significant changes in the total work level (TW) in the 10 s anaerobic exercise during the circadian cycle (*F* = 5.283; *η^2^_P_* = 0.150; *p* = 0.029). At 10:00 p.m., the studied men showed a slight increase in this parameter compared to the measurement at 10:00 a.m. The “time × group” interaction was not noted (*p* > 0.05). However, a tendency (0.1 > *p* > 0.05) indicating the mentioned interaction can be observed. In the BG, the values of this parameter measured at 10:00 p.m. were higher (at the limit of significance level, *p* > 0.05) than at 10:00 a.m., while in the CG, they remained at a similar level.

### 3.3. Hormones

The concentration of hormones in BG and CG showed differentiation. In the CG, the concentration of cortisol in the blood collected at 10:00 a.m. before exercise (191.50 ± 19.94 ng·mL^−1^) was, on average, 58.31 ng·mL^−1^ higher than in the BG (133.19 ± 32.27 ng·mL^−1^). The observed differences are statistically significant (*Z* = −4.602; *p* < 0.001). The concentration of testosterone for the CG collected at this time (4.89 ± 3.68 ng·mL^−1^) was, on average, 0.18 ng·mL^−1^ higher than in the BG (4.71 ± 0.67 ng·mL^−1^); however, the results did not differ significantly. The concentration of the growth hormone (0.27 ± 0.33 ng·mL^−1^) was, on average, about 17 ng·mL^−1^ higher in the CG than the BG (0.10 ± 0.05 ng·mL^−1^); nonetheless, the difference was not statistically significant.

The melatonin concentration at 10:00 a.m. in the BG (44.10 ± 12.19 ng·mL^−1^) was, on average, 7.09 ng·mL^−1^ lower than the concentration noted in the CG (51.19 ± 21.91 ng·mL^−1^). The Mann–Whitney U test results indicate the statistical significance of this difference (*Z* = −4.825, *p* < 0.001) (Table 2).

Treating all subjects as one sample, the results of MANOVA showed a statistically significant decrease in cortisol at 10:00 p.m. compared to the measurement at 10:00 a.m. (*F* = 503.222; *η^2^_P_* = 0.944; *p* < 0.001). In addition, there was a significant “time × group” interaction (*F* = 28.804; *η^2^_P_* = 0.490; *p* < 0.001). In the blind group, the concentration of C was reduced by 88.44 ng·mL^−1^ (*Z* = −3.517; *p* < 0.001) and in the CG, there was a decrease by 144.06 ng·mL^−1^ (*Z* = −3.517; *p* < 0.001).

The results of MANOVA also indicated a statistically significant decrease in testosterone concentration at 10:00 p.m. compared to the measurement at 10:00 a.m. by 0.63 ng·mL^−1^ (*F* = 6.956; *η^2^_P_* = 0.188; *p* = 0.013). However, there was no significant “time × group” interaction for any of the measurements at 10:00 a.m. or 10:00 p.m. (*p* > 0.05). A statistically significant increase in the concentration of the growth hormone at 10:00 p.m. was also demonstrated in comparison to the measurement at 10:00 a.m. (*F* = 32.043; *η^2^_P_* = 0.516; *p* < 0.001). Additionally, there was a significant “time × group” interaction *F* = 31.452; *η^2^_P_* = 0.512; *p* < 0.001. In the BG, the growth hormone concentration increased by 0.01 ng/mL (the change was not statistically significant *p* > 0.05). In contrast, in the CG, the concentration of this hormone increased by 1.34 ng·mL^−1^ (*Z* = −3.413; *p* = 0.001).

Treating the groups (BG + CG) collectively, the results of MANOVA indicated a statistically significant increase in melatonin concentration at 10:00 p.m. compared to the measurement at 10:00 a.m. (*F* = 32.043; *η^2^_P_* = 0.516; *p* = 0.038). In addition, there was a significant “time × group” interaction (measurements performed prior to exercise—*F* = 21.384; *η^2^_P_* = 0.416; *p* < 0.001). In the BG, the melatonin concentration (M) decreased by 7.09 ng·mL^−1^ (statistically significant change *p* < 0.05), in the CG, there was an increase by 19.63 ng·mL^−1^ (*Z* = −3.516; *p <* 0.001).

### 3.4. Correlations

The results of the analysis for measurements from 10:00 a.m. in the BG indicate a lack of dependence between TW and PP and the assayed hormones (*p* > 0.05). However, in the CG, significant correlations between T and PP (r_s_ = 0.580, *p* = 0.019) were indicated. The observed correlation is high and positive. There were no statistically significant relationships (*p* > 0.05) between the remaining variables and the studied hormones measurements from 10:00 a.m. (Table 3).

In the test from 10:00 p.m., differences in correlations were observed compared to 10:00 a.m. However, there were no significant correlations between the anaerobic test indices (PP and TW) and hormone levels in the CG (*p* > 0.05), while in the BG, there were significant relationships between: GH and PP (r_s_ = −0.649; *p* = 0.007). The observed correlation is high and negative, which means that in blind people, the higher the total power values, the lower the concentration of the growth hormone. A similar correlation was found between T and PP (r_s_ = −0.537; *p* = 0.032), which is high and negative, meaning that in blind individuals, the higher the total power values, the lower the testosterone levels. A correlation was also found between M (r_s_ = −0.735, *p* = 0.001) and TW (r_s_ = −0.680, *p* = 0.004) and PP (r_s_ = −0.606; *p* = 0.013). In all of the cases, the observed correlations are high or very high and negative (Table 4).

## 4. Discussion

The human population has a wide range of diurnal chronotypes, with early types (early birds) at one end of the spectrum and late types (owls) at the other. The chronotype is influenced by individual genetics, development, and exposure to light, dawn, and dusk. In terms of genetics, clock gene mutations can explain some differences in the chronotype. In recent large-scale genomic studies, variants have been identified in several clock-related loci [3,4], in particular, underlying mornings in the general population. Different chronotypes usually change sleep patterns to account for both social needs and the daily clock. The method of shaping the characteristic circadian rhythm for each person is defined by the chronotype: morning or evening, designating the time of the day of the greatest and the smallest activity of an organism. A significant percentage of blind people without light perception is characterised by circadian rhythm disturbances. The high incidence of abnormal circadian rhythms in blind people underlines the significance of the light-dark cycle as an important environmental synchroniser for the human circadian system [16].

Determining the time of the day in which physical fitness is the best is very important from the point of view of sports physiology for both healthy and blind athletes. It gives the opportunity to plan an athlete’s training in order to be able to maximally use the exercise performance and effectively influence the achieved results. At the same time, the knowledge of the coach and athlete regarding the ways of modifying the biological rhythm allows a chance for better preparation of the athlete to perform the effort in unfavourable conditions, e.g., change in the hours of sleep resulting from the change in time zone. Appropriately conducted sports training aimed at modifying circadian rhythm can prepare the athlete for the future time change, minimising the negative effects of jet lag and, thus, positively affecting the final sports outcome of an athlete. Studies regarding the influence of circadian rhythm on the development of the anaerobic performance were most often carried out at two selected times of the day, i.e., in the morning at 6:00, 7:00 or 10:00 a.m., and late afternoon/evening at 5:00 or 6:00 p.m. [10,17,18,19,20,21,22,23]. The study conducted four times at 2:00 a.m., 10:00 a.m., 6:00 p.m., and 10:00 p.m. is rare. We have found that the time of day has a significant impact on the exercise capacity, with the highest efficiency is in the afternoon. For example, the results of peak power and body temperature are significantly higher for tests at 6:00 p.m. than at 2:00 a.m. Souissi et al. [24], in the Wingate test, noted significantly higher PP and TW values at 6 pm than at 06:00 a.m. Additionally, Abedelmalek et al. [22] drew attention to the inter-racial differences between athletes in the daily level of Wingate test indices and showed that athletes from southern Africa achieved significantly better results compared to Tunisians. Tyka et al. [25] showed that the highest values of PP, TW, and body temperature occur at 6:00 p.m. and the lowest at night (2:00 a.m.). The highest peak power in the afternoon is consistent with the highest body temperature. However, this was not reflected in the Kin-Isler study [26], in which, although the highest Tre was recorded at 5:00 p.m., the peak power was recorded at 1:00 p.m.

The conclusions from the study by Lericollais et al. [19] seem to be interesting regarding the effect of wakefulness disturbances due to lack of sleep at night on the results of the Wingate test carried out the following morning. It turns out that the results achieved in the test in the morning were not dependent on the partial or complete lack of sleep on the night before exercise. Nevertheless, the tests were not repeated at other times of the day, so it was not shown how sleep disorders affect the exercise capacity depending on circadian rhythm. On the other hand, research by Souissi et al. [27] showed that in judo athletes, sleep disorders in the first or second half of the night caused the disappearance of diurnal periodisation of muscle strength and power during the short-term exercise performed the next day between 9:00 a.m. and 4:00 p.m. and that sleep disturbances in the second half of the night significantly decreased muscle strength and power analysed during the exercise at 4:00 p.m.

Testing by empirical means of modifying the circadian rhythm through the use of targeted training at the same time is an interesting issue among sports physiologists. Such an attempt was made, among others, by Souissi et al. [28], Blonc et al. [29], Chtourou et al. [30], analysing the impact of a several-week training programme (from 3 to 8 weeks) on exercise capacity carried out at the same time of the day each time. The research was conducted among two research groups, the morning one performing training at 7:00 a.m., and the afternoon group, which trained at 5:00 p.m. The level of anaerobic indices was examined before and after several weeks of training. The results of studies by Souissi et al. [28] and Chtourou et al. [30] showed that the daily rhythm of muscle strength and strength was disturbed in the case of people training in the morning. They improved power in the morning to such an extent that they reached the level characteristic for afternoon hours. At the same time, the training effect resulting in an anaerobic capacity increase was observed in both the morning and evening groups. In turn, the results of research by Blonc et al. [29] showed no modification of the circadian values of anaerobic indices as a result of the applied training. However, there was a training effect, and improvement of the results achieved at both examined hours was by about 5–6%. The study by Racinais et al. [31] regarded measurements of muscle power generated during two types of vertical jumps (squat jump, countermovement jump) and an effort on a cycloergometer in the morning (7:00 a.m.) and afternoon (5:00 p.m.), conducted in thermoneutral conditions and at an elevated ambient temperature. In thermoneutral conditions, they showed characteristic daily variability with higher power values achieved in the afternoon. However, at an elevated ambient temperature, they observed deterioration of the obtained results and disappearance of daily variation in the level of this variable. At the same time, they showed that the internal and surface temperature of the body depends on circadian rhythm and ambient temperature. In studies on diurnal variability of anaerobic performance, the effect of applying different warm-up durations was analysed by Racinais et al. [31], Souissi et al. [32], or Chaâri et al. [33]. The decrease in the achieved power values at elevated ambient temperature is confirmed by Tyka et al. [8].

Kanaley et al. [34] showed that the time of day does not affect the post-exercise secretion of the growth hormone. However, it is worth bearing in mind that while this hormone shows circadian rhythm at rest, both physical effort and sleep are strong stimuli that can reduce daily differences in growth hormone secretion [35]. The circadian rhythm of melatonin secretion is caused by changes in light throughout the day [36]. The M concentration during the day is very low. It starts to increase around 9:00/10:00 p.m. and reaches its highest values between 1:00 a.m. and 4:00 a.m. [37,38,39]. Melatonin significantly affects the work of the internal biological clock [37]. In our study, it was found that the pre-dose hormone concentration in the blood serum varied throughout the day, with statistically significant differences between melatonin and cortisol. There was no difference in testosterone or growth hormone levels. Wideman et al. [40] suggested that both aerobic and strength exercises significantly increase the release of growth hormones. In studies, most researchers have observed that the release of the growth hormone increases significantly due to intense exercise [41,42,43]. This was also confirmed in the studies by Pritzlaff et al. [44,45], in which the greater release of growth hormone was observed along with the increase in the intensity of the effort at the lactate threshold and above this threshold. High interest in daily changes of adrenocortical hormone secretion, including cortisol, is associated with the assumption that this rhythm acts as a synchroniser regarding the endogenous rhythms of many systemic functions. The highest blood cortisol levels are found in most people between 6:00 a.m. and 8:00 a.m. in the morning, and the lowest in the time range between 8:00 p.m. and 4:00 a.m. [46]. The results of a study by Kanaley et al. [34] confirmed the differential release of C after exercise depending on the time of day. Obmiński and Sitkowski [47] showed that the magnitude of the secretion of cortisol after the maximal effort depends on its duration. However, the authors suggested that diet, amount and quality of sleep, previous physical activity, and body composition may be factors interfering with the diurnal periodic release of this hormone. The results of our previous studies confirm that exercise at elevated ambient temperatures compensated for the periodisation of the cortisol, testosterone, and growth hormone concentrations in blood serum observed at rest. These changes did not only affect melatonin. This indicates the possibility of reducing daily fluctuations in the secretion of hormones by exercise. In athletes, especially during intense workouts, testosterone is one of the most frequently analysed steroid hormones. T plays an important role in regulating exercise metabolism, taking the process of structural protein recovery into account [48]. Despite the numerous although fragmentary studies, research on the rhythm of testosterone secretion is still not complete. Our study managed to observe the highest T concentration around 8:00 a.m. in the morning and the lowest at around 2:00 p.m. at night [49].

The results of our study showed that in both groups (CG; BG), PP in the test at 10:00 a.m. was significantly higher than at 10:00 p.m. (*F* = 6.679; *η^2^_P_* = 0.182; *p* = 0.015). The observed differences were mainly influenced by the results of men from the BG who had an increase in PP by W kg^−1^, while there were no significant changes in this parameter in subjects from CG (interaction time × group: *F* = 5.945; *η^2^_P_* = 0.165; *p* = 0.021).

The results of the analysis carried out for measurements at 10:00 a.m. in the BG did not show dependence between TW and PP and the studied hormones (*p* > 0.05). In CG, however, significant correlations between testosterone and total work (r_s_ = 0.580; *p* = 0.019) were indicated, meaning that along with the increase in this hormone, the amount of anaerobic work also increased, which is logical substantiation. However, in the tests from 10:00 p.m., there was a significant variation in correlations compared to 10:00 a.m. In CG, there were no significant relationships between TW or PP and the hormones studied in CG (*p* > 0.05), while in BG, there were significant relationships. A negative correlation was found between GH and PP (r_s_ = −0.649; *p* = 0.007), T and PP (r_s_ = −0.537; *p* = 0.032) as well as M (r_s_ = −0.735; *p* = 0.001), TW (r_s_ = −0.680; *p* = 0.004) and PP (r_s_ = −0.606; *p* = 0.013). This means that in the BG, the higher the results of peak power and total work, the lower the concentration of GH, T and M. In all cases, the observed correlation was high or very high and negative.

### Limitation of the Study

In this study, we did not assess nutrition. Nutritional status may affect the muscle capacity to produce force and the nervous system to stimulate the working muscles. During the study, the participants were asked to maintain their current diet and physical activity.

## 5. Conclusions

In blind men, the hormonal response is not determined by the photoperiod as in the case of the controls. It is especially interesting to learn about the anaerobic performance at different times of the day. In the BG group at 10:00 p.m., the PP and TW results were significantly higher than at 10:00 a.m. and were negatively correlated with M, which was not observed in the CG. This may suggest that the higher level of anaerobic power in BG at 10:00 p.m. is associated with a lower concentration of M at this time. However, despite statistically significant differences in M concentration in CG at 10:00 p.m. and 10:00 a.m., no changes in PP or TW levels were noted. This may suggest the influence of other factors on circadian variability of the anaerobic performance.

## Figures and Tables

**Table 1:** Peak power and total work in anaerobic effort in blind men.

Indicator	Hour	Group	M ± SD	Min	Max	Percentile		
						25	50	75
PP (W kg^−1^)	10:00 a.m.	BG	9.90 ± 1.88	6.19	11.93	8.73	10.63	11.53
		CG	12.6 ± 0.70	11.33	13.89	12.25	12.55	12.98
	10:00 p.m.	BG	10.6 ± 1.79	7.21	13.44	9.27	10.97	11.36
		CG	12.6 ± 0.63	11.66	13.50	11.97	12.61	13.26
TW (J kg^−1^)	10:00 a.m.	BG	87.1 ± 14.08	59.0	105.0	78.5	90.0	99.8
		CG	113.4 ± 4.95	101.0	119.0	110.0	111.0	114.8
	10:00 p.m.	BG	91.4 ± 12.48	65.0	109.0	85.3	96.0	99.8
		CG	111.4 ± 3.95	106.0	118.0	108.5	112.0	115.0

BG—blind men; CG—control group; TW—total work; PP—peak power.

**Table 2:** Concentrations of cortisol, testosterone, growth hormone, and melatonin collected before the exercise in blind men and in the control group.

Hormone	Hour	Group	M ± SD	Min	Max	Percentile		
						25	50	75
C (ng·mL^−1^)	10:00 a.m.	BG	133.19 ± 37.27	69.0	176.0	95.8	144.0	168.8
		CG	192.50 ± 19.94	171	241	176.3	187.0	199.0
	10:00 p.m.	BG	44.70 ± 21.63	16.0	90	24.5	42.0	56.8
		CG	74.40 ± 15.30	29.9	90	38.3	44.0	51.3
T (ng·mL^−1^)	10:00 a.m.	BG	4.71 ± 0.67	3.45	5.41	3.99	4.93	5.24
		CG	4.89 ± 3.68	1.46	10.92	1.71	3.33	8.23
	10:00 p.m.	BG	4.38 ± 0.64	3.33	5.51	3.92	4.28	4.91
		CG	3.96 ± 2.39	1.43	9.77	2.00	3.29	5.73
GH (ng·mL^−1^)	10:00 a.m.	BG	0.10 ± 0.05	0.02	0.17	0.06	0.10	0.15
		CG	0.27 ± 0.23	0.02	0.95	0.03	0.12	0.54
	10:00 p.m.	BG	0.11 ± 0.05	0.01	0.20	0.07	0.10	0.14
		CG	1.61 ± 1.02	0.21	3.87	0.82	1.69	2.17
M (ng·mL^−1^)	10:00 a.m.	BG	51.19 ± 21.99	21.96	93.53	35.45	44.17	67.72
		CG	2.72 ± 1.36	1.36	5.20	1.44	2.62	3.64
	10.00 p.m.	BG	44.1 ± 12.19	12.19	63.10	33.45	44.52	55.11
		CG	23.35 ± 12.91	12.91	54.49	12.95	18.61	25.19

BG—blind men; CG—control group; C—cortisol; GH—growth hormone; T—testosterone; M—melatonin.

**Table 3:** Results of the Spearman’s signed-rank correlation test examining the relationship between total work and peak power and the hormones measurements from 10:00 a.m.

		BG (*N* = 16)				CG (*N* = 16)			
		C	GH	T	M	C	GH	T	M
TW (J kg^−1^)	r_s_	0.097	−0.330	−0.200	0.423	−0.182	−0.297	0.580 *	−0.155
	*p*	0.722	0.212	0.458	0.103	0.500	0.263	0.019	0.567
PP (W kg^−1^)	r_s_	0.068	−0.154	−0.396	0.149	0.010	−0.210	0.350	−0.093
	*p*	0.803	0.569	0.129	0.583	0.970	0.435	0.184	0.733

BG—blind men; CG—control group; C—cortisol; GH—growth hormone; T—testosterone; M—melatonin; TW—total work; PP—peak power; *—correlation significant at 0.05.

**Table 4:** Results of the Spearman’s signed-rank correlation test examining the relationship between total work and peak power and the hormones measurements from 10:00 p.m.

		BG (*N* = 16)				CG (*N* = 16)			
		C	GH	T	M	C	GH	T	M
TW (J kg^−1^)	r_s_	−0.133	−0.590 *	−0.352	−0.680 **	−0.036	0.272	0.202	−0.333
	*p*	0.625	0.016	0.182	0.004	0.896	0.309	0.453	0.208
PP (W kg^−1^)	r_s_	0.029	−0.649 **	−0.537 *	−0.606 *	0.037	0.203	0.171	−0.274
	*p*	0.914	0.007	0.032	0.013	0.892	0.451	0.528	0.305

BG—blind men; CG—control group; C—cortisol; GH—growth hormone; T—testosterone; M—melatonin; TW—total work; PP—peak power; *—correlation significant at 0.05; **—correlation significant at 0.01.

## Data Availability

Not applicable.
